# Retraction: Radiological and Functional Outcome of Medial Epicondyle Fracture Treated Surgically in Children and Adolescents: A Retrospective Study

**DOI:** 10.7759/cureus.r9

**Published:** 2017-12-13

**Authors:** Sagar Panthi, Kishor Khatri, Krishna Kharel, Raju Vaishya, Amit Kumar Agarwal, Vipul Vijay

**Affiliations:** 1 Orthopaedics and Trauma Surgeon, Nepalgunj Medical College and Teaching Hospital; 2 Orthopaedics and Trauma Surgeon, Lumbini Zonal Hospital; 3 Orthopaedics, Tilottama Hospital; 4 Department of Orthopedics, Indraprastha Apollo Hospital, New Delhi

This article has been retracted at the request of the Editor-in-Chief and the authors.

The authors have plagiarized the majority of this article as it previously appeared in the The Journal of Lumbini Medical College: Joshi RR, Sundararaj GD. Clinical results of surgically treated medial humeral epicondylar apophyseal avulsion injury in children and adolescent. Journal of Lumbini Medical College. 2014;2(2):31-6. 10.22502/jlmc.v2i2.54.

A key condition of article submission is that authors must explicitly declare that their work is original and has not appeared in a publication elsewhere. Re-use of any data must be appropriately cited. As such this article represents a severe abuse of the scientific publishing system. The Cureus Journal of Medical Science takes a very strong view on this matter and we apologize that this was not detected during the submission process. The relevant author institutions and departments have been notified.

